# Seizure in Indonesian Glioma Patients: Associated Risk Factors and Impact on Survival

**DOI:** 10.31557/APJCP.2021.22.3.691

**Published:** 2021-03

**Authors:** Rachmat Andi Hartanto, Ery Kus Dwianingsih, Andre Stefanus Panggabean, Adiguno Suryo Wicaksono, Kusumo Dananjoyo, Ahmad Asmedi, Rusdy Ghazali Malueka

**Affiliations:** 1 *Division of Neurosurgery, Department of Surgery, Faculty of Medicine, Public Health, and Nursing, Universitas Gadjah Mada, Dr. Sardjito General Hospital, Yogyakarta, Indonesia. *; 2 *Department of Anatomical Pathology, Faculty of Medicine, Public Health, and Nursing, Universitas Gadjah Mada, Dr. Sardjito General Hospital, Yogyakarta, Indonesia. *; 3 *Neurology Department, Faculty of Medicine, Public Health, and Nursing, Universitas Gadjah Mada, Dr Sardjito General Hospital, Yogyakarta, Indonesia. *

**Keywords:** Glioma, seizure, risk factors, survival

## Abstract

**Objective:**

Seizure is commonly found in patients with glioma. This study aimed to find risk factors for seizures in Indonesian patients with glioma. We also sought to determine the association between seizure and survival in this patient population.

**Methods:**

Patients with glioma were enrolled from the Dr. Sardjito General Hospital and other hospitals in Yogyakarta Province, Indonesia. Detailed demographic and clinical data were collected from medical records. DNA extraction and polymerase chain reaction (PCR) were performed to detect *IDH1* mutation. Tumor tissue samples were stained by hematoxylin-eosin and classified according to the 2016 World Health Organization (WHO) classification of central nervous system (CNS) tumors. Expression of *Ki-67* was detected by immunohistochemistry staining. Survival data were also collected.

**Results:**

In total, 107 patients were included in the analysis. Age, gender, history of smoking, tumor side, tumor grade, *Ki-67* expression, and *IDH1* mutation were not associated with seizure. Tumors involving the frontal lobe (p=0.037) and oligodendroglioma histology (p=0.031) were associated with the development of seizures in this study. However, multivariate analysis showed that only oligodendrogial histology was associated with seizure [p=0.032, odds ratio (OR) = 4.77, 95% confidence interval (CI) = 1.146-19.822]. Patients with seizures have significantly longer median overall survival than patients without seizures (69.3±25.01 vs. 10.6±6.14 months, respectively, p=0.04).

**Conclusion:**

This study showed that seizure in patients with glioma in Indonesia is associated with frontal lobe location and oligodendroglioma histology. Patients with seizures also have significantly longer overall survival.

## Introduction

Seizure is commonly found in patients with brain tumors. Hughlings Jackson first described glioma-related seizures in 1882 (Samudra et al., 2019). Seizure can affect the patient’s quality of life due to morbidities and deterioration in cognitive function (Englot et al., 2012). The prevalence of seizures in patients with brain tumors ranges from 30% to 100%, depending on the tumor type. The most common brain tumors that develop seizures are neuroglial tumors and gliomas (Van Breemen et al., 2007). Previous studies showed that 30% to 50% of patients with brain tumors would develop seizure as their first clinical sign, whereas 10% to 30% of the patients will experience seizure later during the disease course (Van Breemen et al., 2007).

The development of seizures in patients with brain tumors depends on many risk factors, such as age, gender, tumor type, tumor location, tumor growth and size, number of tumors, time to histological diagnosis, and increased intracranial pressure. Seizures in brain tumors are commonly found at presentation in patients who are younger than 50 years of age (Moots et al., 1995). The average age at presentation is 15 years for dysembryoplastic neuroepithelial tumor (DNET), 17 to 21 years for gangliogliomas, 38 to 40 years for low-grade gliomas (LGGs), and 60 years for glioblastomas (GBMs) (Compton et al., 2012; Kerkhof et al., 2013; Thom et al., 2011; Van Breemen et al., 2009).

Male gender is also associated with a higher incidence of seizure in brain tumors. In a large study performed by Pallud (2014), 1,355 of 1,509 patients with brain tumors experiencing seizures were male. The study from Bernttson (2018) also reported that glioma occurred more frequently in males than females, 59% and 41%, respectively.

Tumor types play a role in the development of seizures in patients with brain tumors. Although any brain tumor can cause seizures, neuroepithelial tumors are most commonly associated with seizure development. They are divided into neuroglial tumors, low-grade gliomas (LGGs), and high-grade gliomas (HGGs). The neuroglial tumors can further be divided into dysembryoplastic neuroepitheliomas (DNETs) and gangliogliomas. Seizures develop in almost all patients with DNET and in approximately 80% to 90% of those with ganglioglioma. LGGs [World Health Organization (WHO) grade II gliomas consist of astrocytomas, oligodendrogliomas, and oligoastrocytomas] are considered more epileptogenic than HGGs (grade III anaplastic astrocytoma and grade IV GBM). Seizure frequency varies between 65% to 85% in LGGs, 30% to 50% in HGGs, and 15% to 20% in brain metastases (Kerkhof et al., 2013; Vecht and Wilms, 2010). Seizures are more frequent in secondary GBMs, which progress from LGGs instead of primary GBMs, which arise de novo (Rosati et al., 2009). The mechanism of seizures in neuroglial tumors are the changes in the local neurotransmitter levels and association with structural abnormalities in the brain, whereas seizures in diffuse LGGs are caused by denervation hypersensitivity (deafferentation of the nearby brain cortex) (Kurzwelly et al., 2010). 

The location of the brain tumor is an important factor for the development of seizures. Seizures are more common in frontal, temporal, and parietal lobe tumors compared with those in the occipital lobe (Moots et al., 1995; Rudà et al., 2010; Sirven et al., 2004). Tumors that are close to the functional area and located in the supratentorial region, insular cortex, and superficial cortex have a higher risk of seizures (Englot et al., 2016; Pallud et al., 2014). Whereas infratentorial and sellar tumors infrequently produce seizures unless they expand into the cerebral hemisphere (Van Breemen et al., 2007). 

Slower growing tumors and smaller tumors are associated with a higher risk for seizure than larger and rapidly growing tumors. The smaller tumors develop focal and remote cell changes that are associated with epileptogenesis. Whereas rapidly progressive tumors might induce seizure through abrupt tissue damage such as deposition of hemosiderin and tissue necrosis. The seizure predilection of HGGs, which are rapidly progressive tumors in brain white matter where epileptogenesis rarely happens, is low. Moreover, patients with malignant brain tumors do not survive long enough to develop seizures (Cascino, 1990; Chaichana et al., 2009; Englot et al., 2016; Riva, 2005).

Seizures are also more common in patients with multiple or multifocal tumors than in those with a solitary tumor (Lee et al., 2010; Moots et al., 1995; Sperling and Ko, 2006). The other factors that might increase the risk of seizure developments are increased intracranial pressure and longer time to histological diagnosis (Pallud et al., 2014).

The tumor molecular characteristic has also been associated with the development of seizures in glioma. A meta-analysis showed an association between* IDH1 *mutation and higher preoperative seizure incidence in LGG (Li et al., 2018). Other studies have also shown a significant association between *IDH1* mutation and seizure regardless of grade (Chen et al., 2017), and also in GBM (Toledo et al., 2017). Mutant isocitrate dehydrogenase 1 produces d-2-hydroxyglutarate (D2HG), which is structurally similar to glutamate, an excitatory neurotransmitter. Glioma patients with *IDH1* mutation are more likely to have seizures because of the effect of D2HG in mimicking glutamate activity on the N-methyl-D-aspartate (NMDA) receptor, which will result in increased neuronal activity (Chen et al., 2017).

Seizure in patients with glioma has been associated with survival. Interestingly, glioma patients with seizures were shown to have longer survival (Toledo et al., 2017; Yang et al., 2014). This is probably related to glioma grading. As mentioned before, patients with LGG are more likely to have seizures while also having better survival outcomes than patients with HGG (Berntsson et al., 2018). 

The risk factors for seizure and its association with survival in patients with glioma have never been studied in Indonesia. This study aimed to find risk factors for seizures in Indonesian patients with glioma. We also sought to determine the association between seizure and survival in this population. 

## Materials and Methods

*Patients and samples*

The patients with glioma in this study were enrolled from Dr. Sardjito General Hospital and other hospitals in Yogyakarta Province, Indonesia. This study was approved by the Institutional Review Board (IRB), Faculty of Medicine, Public Health, and Nursing, Gadjah Mada University, Indonesia. Written informed consent was obtained from the patients themselves or from a family member. Tumor tissue samples were stained with hematoxylin-eosin, assessed by an experienced neuropathologist, and classified according to the 2016 WHO classification of central nervous system (CNS) tumors. Detailed demographic and clinical data were collected from medical records.

*DNA extraction*

Genomic DNA was extracted from fresh tumor tissue specimens or from formalin-fixed paraffin-embedded (FFPE) tumor tissue. DNA from fresh glioma tissue was extracted using the Quick DNA FFPE MiniPrep Kit (Zymo Research, USA). DNA from FFPE tissue specimens was isolated using the QIAamp DNA FFPE Tissue Kit (QIAGEN, Cat. #56404, Hilden, Germany) according to the manufacturer’s instructions. 

*Identification of IDH1 mutation status*

Polymerase chain reaction (PCR) was performed using forward (5’-ACC AAA TGG CAC CAT ACG A-3’) and reverse (5’-GCA AAA TCA CAT TAT TGC CAA C-3’) primers to amplify codon R132 of the exon 4 of the *IDH1* gene (Arita et al., 2014). PCR reactions were performed in a volume of 25 µL containing 2 µL of genomic DNA, 3.45 µL of Taq DNA Polymerase (Invitrogen, Thermo Fisher Scientific, Cat. #10342020, Waltham, MA, USA), 0.2 µL of dNTP (Thermo Scientific, Thermo Fisher Scientific, Cat. #R0191, Waltham, MA, USA), and 1 µL of each primer. Conditions for PCR cycling were as follows: initial denaturation at 95^o^C for 2 minutes followed by 40 cycles of denaturation at 95^o^C for 30 seconds, annealing at 53^o^C for 30 seconds, and extension at 72^o^C for 2.5 minutes. The PCR products were sequenced using the BigDye Terminator v3.1 Cycle Sequencing Kit (Applied Biosystem, Thermo Fisher Scientific, Cat. #4337455, Waltham, MA, USA). Each sample was defined as positive or negative for *IDH1* gene mutation based on the sequencing results.

*Ki-67 expression status*

Expression of Ki-67 was detected by immunohistochemistry staining using an antibody for Ki-67 (SP6, rabbit monoclonal antibody, Biocare Medical, Cat. #OAI325T60, Pacheco, CA, USA). A trained pathologist examined the percentage of positively stained tumor cells (Zhang et al., 2013). 

*Survival data collection*

The survival data were collected in the outpatient clinic during patients’ visits, in the ward during patient hospitalization periods, and through phone calls or home visits. The overall survival (OS) was calculated as the time between the first surgical intervention and death or last follow-up (for censored cases). 

*Statistical analysis*

The association between seizure and various risk factors was analyzed. A T-test or Mann-Whitney test was used to compare the mean of numerical data between patients with and without seizure. Meanwhile, the chi-square or Fisher exact test was used to analyze categorical variables. Multivariate analysis using logistic regression with the backward method was performed to identify variables independently associated with seizure. Survival distributions for each group were plotted using the Kaplan-Meier method and compared statistically using the log-rank test. 

## Results

In total, 107 patients were included in this study, consisting of 60 males and 47 females (Table 1). The mean age was 39.77±19.04 years old. Patients with seizures were younger compared to patients without seizures (36.32±17.77 vs. 40.82±19.39 years old). However, this difference was not statistically significant (p=0.173). Likewise, there was no difference in sex distribution nor history of smoking between both groups. The most common location of the glioma found in this study was in the parietal lobe (48.8%), followed by the temporal lobe and frontal lobe. Tumors involving the frontal lobe were more likely to cause seizure (p=0.037, frontal lobe involved vs. not involved). 

According to histological type, the most common type of glial tumors found in this study was astrocytoma (85%), followed by oligodendroglioma (9%), ependymoma (8%), and oligoastrocytoma (5%). Tumor histology was also associated with seizures. Patients with oligodendroglioma were more likely to have seizures, with 55.56% of patients with this tumor histology having seizures (p=0.031, oligodendroglioma vs. other types). This percentage is much higher compared with the percentage of seizures in astrocytoma (22.35%), oligoastrocytoma (20%), and ependymoma (0%). 

The majority of patients (44%) had grade IV glioma (Glioblastoma), followed by grade II (31.78%), grade III (20.56%), and grade I glioma (6.54%). There was no difference in tumor grading between patients with and without seizure (p=0.406). Mutation in codon 132 of the* IDH1* gene was found in 18 (17.14%) patients. Methylation of the MGMT promoter region was found in 22.4% of patients. Both the *IDH* mutation and *MGMT* methylation status were not associated with seizure in this study (p= 0.128 and 0.46, respectively). Other factors, including tumor side and Ki-67 expression, were also not associated with seizure. 

Multivariate analysis using logistic regression with backward method showed that only oligodendrogial histology was associated with seizure (p=0.032, OR=4.77, 95% CI= 1.146-19.822, Table 2). The analysis showed an odds ratio of 4.77 for oligodendrogial tumors to have seizures, compared to the non-oligodendrogial tumors as reference. 

**Figure 1:**
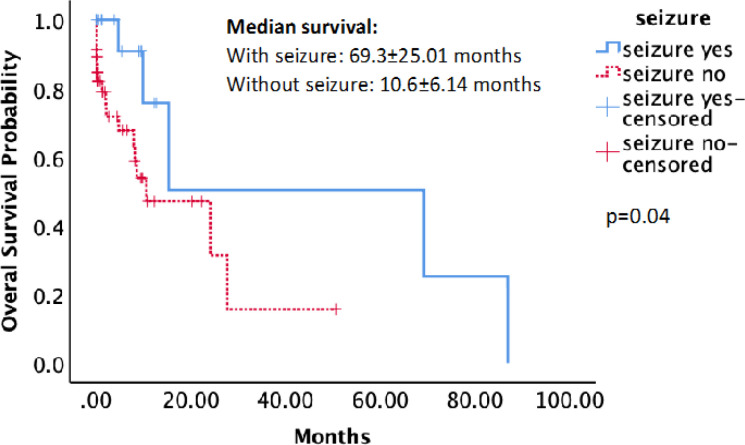
Kaplan-Meier Survival Analysis Demonstrating that Patients with Seizure (Straight Line) had a Significantly Longer Overall Survival (p = 0.04; log-rank Test).

We also analyzed the impact of seizures on overall survival. As shown in Figure 1, patients with seizures have significantly longer median overall survival than patients without seizures (69.3±25.01 vs. 10.6±6.14 months, respectively). A Kaplan-Meier survival analysis using the log-rank method showed that this difference was statistically significant (p=0.04).

**Table 1:** Subject Characteristics (n=107)

Variable	Total (n=107)	With seizure (n=25)	No seizure (n=82)	p-value
Age, mean in years (SD)	39.77(19.04)	36.32(17.77)	40.82 (19.39)	0.173^#^
Sex, n (%)				
Male	60 (56.07)	13 (52)	47 (57.32)	0.639
Female	47 (43.93)	12 (48)	35 (42.68)	
A history of smoking, n (%)				
Yes	27 (28.13)	9 (39.13)	18 (24.66)	0.178
No	69 (71.87)	14 (60.87)	55(75.34)	
Tumor location (include this lobe/not)				
Frontal	29/57	11/10	18/47	0.037
Temporal	41/45	12/9	29/36	0.318
Parietal	42/44	8/13	34/31	0.257
Occipital	9/77	3/18	6/59	0.682^##^
Others	14/72	1/20	13/52	0.172^##^
Tumor side, n (%)				
Left	39 (100)	9 (23.08)	30 (76.92)	0.510
Right	30 (100)	10 (33.33)	20 (66.67)	0.262
Bilateral	7 (100)	1 (14.29)	6 (85.71)	0.668^##^
Histological type, n (%)				
Astrocytoma	85 (100)	19 (22.35)	66 (77.65)	0.627^##^
Oligoastrocytoma	5 (100)	1 (20)	4 (80)	1^##^
Oligodendroglioma	9 (100)	5 (55.56)	4 (44.44)	0.031^##^
Ependymoma	8 (100)	0 (0)	8 (100)	0.194^##^
Grade, n (%)				
Grade I	7 (6.54)	1 (4)	6 (7.32)	0.406^##^
Grade II	34 (31.78)	8 (32)	26 (31.71)	
Grade III	22 (20.56)	8 (32)	14 (17.07)	
Grade IV	44 (41.12)	8 (32)	36 (43.9)	
Combined grade, n (%)				
Grade I & II (low grade)	38 (35.51)	7 (28)	31 (37.8)	0.37
Grade III & IV (high grade)	69 (64.49)	18 (72)	51 (62.2)	
Ki-67, mean (SD)	16.71 (16.54)	16.45 (15.17)	16.81 (17.16)	0.740^#^
IDH1 mutation				
Wild type	87 (82.86)	18 (72)	69 (86.25)	0.128^##^
Mutant	18 (17.14)	7 (28)	11 (13.75)	
MGMT promoter methylation, n (%)				
Methylated	13 (22.41)	4 (30.77)	9 (20)	0.460^##^
Unmethylated	45 (77.59)	9 (69.23)	36 (80)	

^#^, Mann-Whitney U test; ^##^, Fisher exact test; Chi-square tests were performed for the rest of the analyses.

**Table 2:** Multivariate Analysis Using Logistic Regression for Predictors of Preoperative Seizure in Glioma

Variable	Odds ratio	95 % CI	p value
Oligodendroglioma	4.77^#^	1.146-19.822	0.032

^#^, Compared to non oligodendrogial tumor.

## Discussion

Our study showed that factors associated with seizure in glioma patients were frontal lobe location and oligodendroglial histology. This study also showed that patients with seizures had significantly longer survival compared with patients without seizures. 

Seizure has been reported as an important symptom in patients with glioma. One theory on the pathogenesis of tumor-related seizure stated that mechanical compression of the normal tissue by the tumor results in the tissue becoming an epileptic focus following ischemia and hypoxia. Another theory stated that the tumor itself could release chemical factors that change the peritumoral microenvironment into an epileptic focus (Yang et al., 2014). Indeed, astrocytic cells had been shown to be able to generate action potential, which acts as the source of epileptic activity (Bordey and Sontheimer, 1998). Glioma cells have also been shown to release glutamate, which is important in glioma-related seizures (Ye and Sontheimer, 1999).

The percentage of patients with preoperative seizures in our study was 23.36%. This is in line with previous studies showing that 15% to 30% of patients diagnosed with a brain tumor had seizures as the presenting symptoms. A large international case-control study showed that 28.5% of glioma cases reported the diagnosis of seizure, convulsion, or epilepsy (Berntsson et al., 2018). 

Our study did not show a significant association between seizure and gender. Previous studies showed that seizure is more common in male patients compared with female patients. Pallud (2014) showed that 1355 of 1509 patients with brain tumors who had seizures were male. A study by Bernttson (2018) also reported that glioma occurred more frequently in males than females (59% and 41%, respectively).

This study also did not show an association between age and seizure. A previous study showed that seizures in brain tumors are more commonly found in patients younger than 50 years (Moots et al., 1995). However, this average age differed based on tumor histological types. The average age at presentation is 15 years for dysembryoplastic neuroepithelial tumor (DNET), 17 to 21 years for gangliogliomas, 38 to 40 years for LGGs, and 60 years for GBMs (Compton et al., 2012; Kerkhof and Vecht, 2013; Thom et al., 2011; Van Breemen et al., 2009). In our study, the average age of patients with glioma with seizure was 39.77**±** 19.04 years. 

This study confirmed the results of previous studies showing that seizure in patients with glioma is associated with the tumor location. Seizures are more common in the frontal, temporal, and parietal lobes rather than in the occipital lobe (Moots et al., 1995; Rudà et al., 2010; Sirven et al., 2004). Our study showed that tumors in the frontal lobe are associated with higher incidence of preoperative seizure. This is probably related to the histology of the glioma located in the frontal lobe. Oligodendroglial tumors, which are more prone to seizure, are more often located in frontal areas. On the other hand, astrocytomas are more frequently located in temporal or insular areas (Berntsson et al., 2018). Indeed, our study showed that 88.9% of oligodendrogliomas are located in the frontal region. 

Our study showed that the most important predictor for seizure in patients with glioma is histology type. In particular, oligodendroglioma histology is strongly associated with the occurrence of seizure, with an odds ratio (OR) of 4.77 (95% CI: 1.146-19.822, p=0.032). Previous studies had shown conflicting results regarding the role of histology in glioma seizure. A previous study by Yang (2014) did not show a difference in seizure incidence between tumor types. However, other studies showed that oligodendroglial tumors are more frequently associated with seizures compared with astrocytomas. This is in line with previous studies (Berntsson et al., 2018; Chang et al., 2008; Samudra et al., 2019; Sperling and Ko, 2006; Van Breemen et al., 2007). Patients with grade II oligodendroglioma/oligoastrocytoma were reported to be three times more likely to have epileptic seizures compared with GBM patients in a large international case-control study (Berntsson et al., 2018). Our study showed higher OR compared with that study, indicating that glioma patients with oligodendroglioma histology were almost five times more likely to have seizures. This can be explained by the fact that oligodendrogliomas more often involve the cortex; therefore, they are more likely to cause seizures compared with astrocytomas, which tend to be situated in the white matter (Kerkhof et al., 2015; Rudà et al., 2012).

Unlike previous studies, our data did not show a difference in seizure incidence between LGG and HGG (p=0.37). Previous studies have consistently shown that LGG is associated with seizures (Berntsson et al., 2018; Pallud et al., 2014; Sperling and Ko, 2006; Van Breemen et al., 2007). The percentage of seizures in low-grade and slow-growing tumors, such as oligodendrogliomas (70.9%), astrocytomas (58.5%), and meningiomas (36.9%), are higher compared with faster-growing tumors, such as GBM (14% to 29%). This has been explained by the relatively long life expectancy of patients with LGG. The development of seizure focuses in a brain tumor requires a relatively long time interval. A slow-growing tumor might partly isolate a brain region through a mechanical or vascular mechanism, causing chronic deafferentation and disconnection of the circumscribed cortical area, leading to denervation hypersensitivity. This area, therefore, has intrinsic epileptogenic propensity (Berntsson et al., 2018). The short life expectancy of people with malignant tumors may limit the chances for seizures to develop (Sperling and Ko, 2006). Therefore, patients with LGGs, especially oligodendroglioma, are more likely to have seizures compared with patients with HGG (Berntsson et al., 2018). The insignificant association in our study is probably caused by the relatively small number of LGGs in our study (35.5%), with only seven seizure cases among them. 

Our study also did not show an association between *IDH1* mutation and seizure (p=0.128). This is different from previous studies showing that *IDH1* mutation is associated with seizures. A meta-analysis showed that *IDH1* mutation was associated with a higher preoperative seizure incidence in LGG (Li et al., 2018). Other studies have also shown a significant association between *IDH1 *mutation and seizure regardless of grade (Chen et al., 2017), and also in GBM (Toledo et al., 2017). We did find a higher proportion of the IDH1 mutant group with seizure compared with the IDH wild-type group (38.89% vs. 20.69%); however, this difference was not statistically significant (p=0.128), probably because of the small number of patients with* IDH1* mutation in our study (18 patients). 

We found significantly longer overall survival in seizure patients compared with patients without seizure (median: 69.3±25.01 months vs. 10.6±6.14 months, respectively, p=0.04). This is consistent with previous studies showing longer survival in glioma patients with seizures (Toledo et al., 2017; Yang et al., 2014). This is probably related to glioma grading. Patients with lower-grade glioma are more likely to have seizures while also having better survival outcomes. Another proposed cause is that lesions with greater cortical involvement are more likely to be associated with seizure. Tumors in the cortical area are more likely to be totally resected, which will result in better overall survival (Berntsson et al., 2018). 

This study has several limitations. The first limitation is the relatively small number of LGGs in our study (35.5%), with only seven seizure cases among them. This is probably the reason why glioma grading was not associated with seizure in this study. The second limitation is the relatively small number of cases with *IDH* mutation in our study, which possibly resulted in the insignificance of association between* IDH1* mutation and seizure. The third limitation is several variables that potentially affect seizure, such as tumor size and the number of tumors, were not analyzed. 

In conclusion, our study showed that seizure in patients with glioma is associated with frontal lobe location and oligodendroglioma histology. Patients with seizures also have significantly longer overall survival. This is the first study determining predictors of preoperative seizure in patients with glioma and the association between seizure and survival in Indonesian patients. 

## Author Contribution Statement

RAH and RGM conceived the presented idea and designed the study. RAH, EKD, and ASP performed the laboratory experiment. RAH, ASW, KD, AA, and RGM collected the samples and provided clinical data. RAH and RGM developed the theory, performed the statistical analysis., and wrote the early draft of the manuscript. All authors discussed the results and contributed to the final manuscript. All projects were supervised by RGM.
